# Regulatory T Cells in Chronic Heart Failure

**DOI:** 10.3389/fimmu.2021.732794

**Published:** 2021-09-22

**Authors:** Yuzhi Lu, Ni Xia, Xiang Cheng

**Affiliations:** ^1^ Department of Cardiology, Union Hospital, Tongji Medical College, Huazhong University of Science and Technology, Wuhan, China; ^2^ Key Laboratory of Biological Targeted Therapy of the Ministry of Education, Union Hospital, Tongji Medical College, Huazhong University of Science and Technology, Wuhan, China

**Keywords:** heart failure, regulatory T cell, immune cell, cardiomyocyte, fibroblast, endothelial cell

## Abstract

Heart failure is a global problem with high hospitalization and mortality rates. Inflammation and immune dysfunction are involved in this disease. Owing to their unique function, regulatory T cells (Tregs) have reacquired attention recently. They participate in immunoregulation and tissue repair in the pathophysiology of heart failure. Tregs are beneficial in heart by suppressing excessive inflammatory responses and promoting stable scar formation in the early stage of heart injury. However, in chronic heart failure, the phenotypes and functions of Tregs changed. They transformed into an antiangiogenic and profibrotic cell type. In this review, we summarized the functions of Tregs in the development of chronic heart failure first. Then, we focused on the interactions between Tregs and their target cells. The target cells of Tregs include immune cells (such as monocytes/macrophages, dendritic cells, T cells, and B cells) and parenchymal cells (such as cardiomyocytes, fibroblasts, and endothelial cells). Next-generation sequencing and gene editing technology make immunotherapy of heart failure possible. So, prospective therapeutic approaches based on Tregs in chronic heart failure had also been evaluated.

## Introduction

Heart failure (HF) is a complex clinical syndrome caused by the progressing of various heart diseases. Structural and functional defects of heart lead to impaired cardiac filling or ejection of blood. Shortness of breath, fluid retention, and fatigue are the classic symptoms of HF (1). It has become a public health problem because of high morbidity, hospitalization, and mortality rates. HF influences more than 37.7 million patients globally (2). Sudden cardiac death and multiple organ dysfunction account for nearly 10% annual mortality in HF patients (3). Therefore, taking effective measures before HF or exploring new strategies to reduce the mortality of HF is urgently needed.

Different ways can be used to classify HF. According to the location of the deficit parts, HF can be divided into left ventricular HF, right ventricular HF, and biventricular HF. Based on the change of ejection fraction value, two types of HF can be classified. It includes HF with preserved ejection fraction (HFpEF) and HF with reduced ejection fraction (HFrEF). The ejection fraction of HFpEF patients is unusually more than 50% or at least over 40%. HFpEF is always accompanied by interstitial fibrosis, thicken ventricular wall, and decreased ventricular compliance. The incidence of HFpEF increases gradually in recent years. However, lacking effective therapeutic drugs in the clinical treatment of HFpEF makes it a poor prognosis. HFrEF usually occurs once a large amount of cardiomyocytes (CMs) is lost, such as in myocardial infarction (MI). The decompensated phase of cardiomyopathy is also manifested with a decreased ejection fraction. Depending on the speed of onset, HF can be divided into acute HF and chronic HF. Multiple factors, such as infection and volume overload, induce the occurrence of acute HF in chronic HF patients and increase the risk of readmission and mortality rate (1). Due to the rapid progress of the disease, the time window for the clinic research of acute HF is inadequate. Rarely had study on immune cells in acute HF been done. In this review, we mainly focused on chronic HF and described the important roles of Tregs in the development of chronic HF.

Ischemic injury, especially MI, accounts for the main reason of chronic HF. The process of MI to HF includes three consecutive but overlapping stages. Massive CMs die when coronary artery is suddenly interrupted. Necrotic cells release debris and induce an inflammation response, which leads to the infiltration of a large number of immune cells and degradation of the extracellular matrix (ECM) (inflammation stage). With the removal of debris and necrotic cells, inflammation is resolved and scar is initially formed (repair stage). In mature stage, cells related to tissue repair are deactivated. At the same time, collagen is cross-linked. These changes promote the formation of stable scar. However, long-term mechanical stress and the activation of the sympathetic nerve and the renin–angiotensin–aldosterone system lead to negative ventricular remodeling and chronic HF (3). Genetic mutation and previous viral or bacterial infection are the main reasons of cardiomyopathy. The release of autoantigens and pathogen-related molecular patterns activate the immune response, which leads to fibrosis and negative ventricular remodeling of the heart.

Inflammation and immune cells participate in both acute heart injury and chronic HF. Inflammatory factors and inflammation-related lectin, such as tumor necrosis factor (TNF)-α, interleukin (IL) -1β, IL-6, lectin 3, are increased in HF patients. Single-cell RNA sequencing of CD45^+^ cells in myocarditis and pressure overload-induced HF mice suggested an immune activation state during HF (4, 5). Most of the clinical trials based on anti-inflammatory or immunoregulatory treatments yet yielded disappointing results, except for IL-1β antibody canakinumab and IL-1 receptor antagonist anakinra in specific populations. For detailed information on these clinical trials, please refer to the review by Van Linthout and Tschöpe (6). These unsatisfactory results remind us of the complexity of immune response after HF. Regulatory T cells (Tregs), standing in the central site in immunomodulation, participate in the process of chronic HF. Here, we concentrated on Tregs and described their interactions with their target cells in HF. The approaches based on Tregs in the treatment of HF had also been referred.

## Regulatory T Cells in Heart Failure and Heart Failure-Related Diseases

Tregs are defined as CD4^+^CD25^hi^Foxp3^+^ cells. Foxp3 (forkhead box P3) is a transcription factor that is necessary for the development and function of Tregs. Dysfunction of Tregs due to *Foxp3* mutation leads to fatal autoimmune diseases. Depending on the different sources, Tregs can be divided into thymus-derived Tregs (tTregs) and peripheral Tregs (pTregs) *in vivo* or induced Tregs (iTregs) *in vitro* (7).

Suppressing excessive immune response and maintaining immune homeostasis and peripheral tolerance are the classic functions of Tregs. Inhibitory cytokines of Tregs, including IL-10, transforming growth factor (TGF)–β, and IL-35, participate in the suppression function of T cells directly (8–10). Molecules on Tregs, such as cytotoxic T lymphocyte-associated antigen 4 (CTLA4) and lymphocyte activation gene 3 (LAG3), also take part in immunomodulation indirectly by interacting with antigen-presenting cells (APCs), especially dendritic cells (DCs) (11, 12). Exoenzyme CD39 or CD39/CD73 on Tregs converts extracellular ATP into adenosine, which suppresses the immune response (13). It is rare but had been described that Tregs played a cytolytic effect by producing granzyme B (14). Recent evidence indicated that Tregs in non-lymphoid tissue obtained specific phenotypes and functions in tissue repair. Tregs in visceral adipose tissue regulated adipocyte metabolism through the transcription factor peroxisome proliferator-activated receptor gamma (15). Analogously, in injured skeletal muscle, Tregs accumulated in the lesion and produced amphiregulin (Areg). Areg promoted the skeletal muscle repair (16). As the Notch signaling ligand, Jagged-1 was highly expressed on skin Tregs, which was necessary for hair regeneration (17). Since then, tissue Tregs had been found in cerebral ischemic injury and acute lung injury (18, 19). Recently, we reported a kind of Tregs that expresses “secreted protein acidic and rich in cysteine” (SPARC) in the hearts of MI mice. They played a protective role in preventing cardiac rupture (20). This result demonstrates that heart Tregs participate in cardiac repair directly. All the results raise the potential protective effects of Tregs on parenchymal cells.

HF is the end stage of almost all cardiovascular diseases. Numerous studies reported that Tregs acted as a protective subset in the early stage of heart injury. Tregs increased in the hearts and mediastinal lymph nodes in mice after acute MI and promoted tissue repair (21, 22). Adoptive transfer of Tregs ameliorated fibrosis and improved mouse heart function after MI (22). In myocardial ischemia–reperfusion (I/R) injury, the similar prevalent role of Tregs had also been reported in mice (23). IL-2 complexes (IL-2Cs) amplified Tregs *in vivo* (24). In ischemic heart injury, IL-2Cs expanded Tregs and maintained heart function in mice (25, 26). Super agonistic anti-CD28 antibody (CD28-SA) and IL-33 amplified Tregs preferentially and improved cardiac contractility in MI mice. They may be used for Treg’s therapy in the future (20, 21, 27). Similarly, infusion of Tregs extenuated cardiac hypertrophy and ventricular remodeling induced by angiotensin II (Ang II). Tregs reduced the mortality of coxsackievirus B3 (CVB3)-induced myocarditis in mice, which reemphasized the protective role of Tregs (28, 29). C-X-C motif chemokine receptor 4 (CXCR4) is essential for Tregs’ accumulation in lesions. POL5551, the antagonist of CXCR4, increased the mobilization and accumulation of Tregs to infarcted heart and improved the prognosis of I/R injury in mice (30). However, in HF mice, Tregs became a Th1-like pro-inflammatory subset and promoted adverse ventricular remodeling (31). These results indicate that Tregs are plastic in phenotype and function in different periods after heart injury. However, what boosts these changes deserves further study.

Clinically, studies reported a positive correlation between a low frequency of Tregs and high risk of cardiovascular disease. It might be used as an independent predictor for rehospitalization in patients with worsening HF (32, 33). Treg’s number decreased in the peripheral blood but significantly increased in the coronary thrombi in acute MI patients. Different from peripheral blood, the T-cell receptors (TCRs) of Tregs in the coronary thrombi showed an oligoclonal characteristic (34–36). A reduced number of Tregs had also been observed in the peripheral blood of patients with acute coronary syndrome, dilated cardiomyopathy (DCM), and ischemic HF (37–40). Increased apoptosis and impaired output of Tregs from the thymus during HF may contribute to the decrease (41). Recently, a clinic study described a positive association between low fraction of circulating Tregs and high mortality in ischemic HFrEF patients (42). Except for the change in Treg number, the suppression function of Tregs on conventional T cells decreased. The secretion of soluble fibrinogen-like protein 2, a novel effector factor of Tregs, was also impaired in ischemic HF patients (43). Aldesleukina is a kind of recombinant human IL-2. A clinical trial using Aldesleukina in patients with stable ischemic heart disease and acute coronary syndrome to evaluate its ability in increasing the number of circulating Tregs has been conducted in 2018. However, the final result has not been reported (TRIAL REGISTRATION NUMBER: NCT03113733).

**Figure 1:**
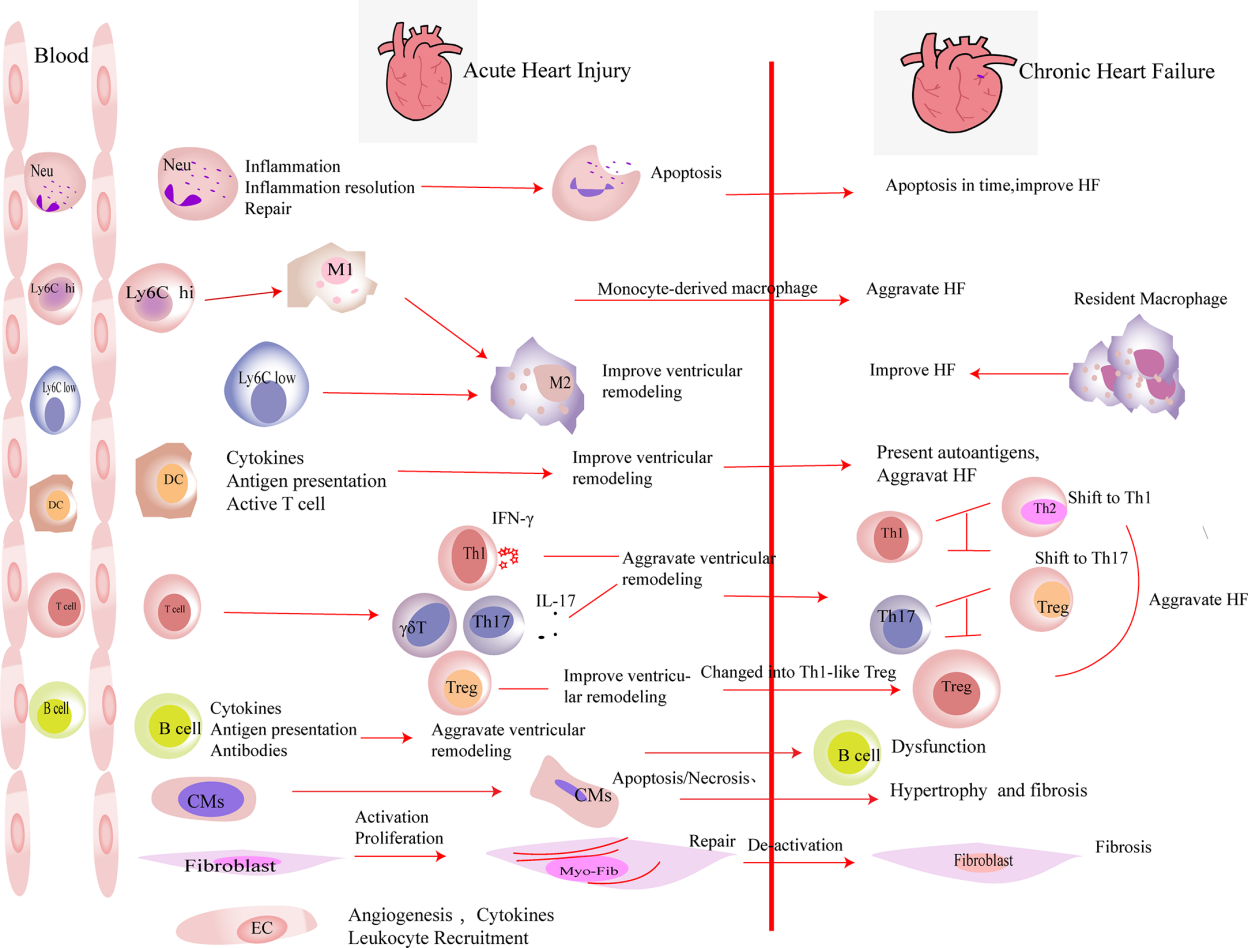
The roles of immune cells and parenchymal cells in acute heart injury and chronic heart failure. We summarized the immune cells that infiltrated the heart in a large number after acute heart injury. Neutrophils (Neu) infiltrate the heart in the very early stage of heart injury. They participate in the inflammation response and then undergo apoptosis, which promote the resolution of inflammation. Neutrophils are indispensable in heart repair after injury. Monocytes (including Ly6C^low^ and Ly6C^hi^ monocytes) accumulate in the heart after acute injury and differentiate into macrophages after phagocytosis of cell debris. M2 polarization of macrophages improves the prognosis of acute heart injury. In heart failure, monocyte-derived macrophages aggravate the damage, while resident macrophages play an anti-inflammatory effect and improve the prognosis of heart failure. DCs participate in the inflammatory response in the early stage of heart injury. They secrete cytokines, present antigens, and activate T cells, which play an important role in heart repair in the early stage of damage. DCs have the ability to activate CD4^+^ T cells and cytotoxic CD8^+^ T cells, which exacerbate HF. T cells play different roles after myocardial infarction. In the early stage of myocardial infarction, they are essential for the repair, while the activated T cells aggravate the damage during heart failure. After myocardial infarction, Th1, Th17, and γδ T cells aggravate ventricular remodeling by producing pro-inflammatory factors. In heart failure, the imbalances of Th1/Th2 and Th17/regulatory T cell (Treg) were observed. Tregs inhibit the inflammation response in the early stage of injury. At the same time, they produce repair-related molecules to promote repair directly. However, in heart failure, Tregs change their phenotype and function and worsen heart failure. B cells produce pro-inflammatory factors in the early stage of injury and recruit monocytes into the heart to aggravate acute heart injury. B cells are dysfunctional in heart failure. The inhibitory function of regulatory B cell (Breg) is damaged. Substantial antibodies and complements are produced by B cells, which aggravate heart failure. A large number of cardiomyocytes (CMs) die in the acute phase of myocardial infarction. They release damage-associated molecular patterns and pro-inflammatory factors. Then, the necrotic CMs are cleared, and extracellular matrix is deposited. CMs’ apoptosis in non-infarct area, which exacerbates interstitial fibrosis. Fibroblasts are activated in the early stage of damage and participate in tissue repair. Then, they are deactivated. The excessive proliferation and activation of fibroblasts and delayed deactivation all aggravate the prognosis of heart failure. Endothelial cells (ECs) are activated in the early stage of injury. They produce molecules that promote the recruitment of leukocytes and participate in the formation of new blood vessels. Their roles in heart failure have not yet been elucidated.

Immune cells and parenchymal cells play important roles in both the early stage of heart injury and the development of HF. Mechanistically, except for producing anti-inflammatory factors, Tregs also participate in HF and HF-related diseases by interacting with immune and parenchymal cells directly. The roles of these related cells in heart had been described in detail in the following subsections (**Figure 1**).

## Regulatory T Cells Interact With Heart Failure-Related Immune Cells

### Regulatory T Cells Interact With Monocytes/Macrophages in Heart Failure

In mice, monocytes include Ly6C^hi^ monocytes and Ly6C^low^ monocytes. Following neutrophils, Ly6C^hi^ monocytes are recruited to the heart in the very early stage of injury. They produce pro-inflammatory factors, such as IL-1, IL-6, and TNF-α. Subsequently, Ly6C^low^ monocytes migrate to the lesion. They secrete anti-inflammatory factors, such as IL-10 (44). By devouring necrotic cells and debris, monocytes transform into macrophages. Macrophages can also be divided into two groups: M1 (classical macrophages) and M2 (alternative macrophages) in mice. M1 macrophages are very efficient in producing toxic intermediates and pro-inflammatory cytokines. In contrast to M1 macrophages, M2 macrophages produce molecules, such as IL-10 and osteopontin. They improve tissue repair by promoting inflammation resolution and angiogenesis (45).

An increased number of monocytes/macrophages had been observed in mice of heart injury. The number of pro-inflammatory CD11b^+^F4/80^+^Gr-1^hi^ monocytes and CD11b^+^F4/80^+^CD206^-^ M1 macrophages increased in the peripheral blood and hearts of ischemic HF mice, respectively (46). Similarly, an accumulation of macrophages in the remote myocardium of MI-related HF in mice was also reported. Furthermore, the researchers found that monocyte’s recruitment from circulation and macrophage’s local proliferation contributed to the expansion of heart macrophages. Inhibiting the recruitment of monocytes improved heart function, which indicates a negative role of monocyte-derived macrophages in the heart (47). However, depletion of macrophages impaired repair and deteriorated ventricular remodeling in cryoinjured hearts of mice (48). Studies based on the distinct lineage of macrophages in heart injury verified the heterogeneous effects of macrophages in mice. It may explain the inconsistent results. MHC^hi^CCR2^-^ and MHCII^low^CCR2^-^ macrophages are resident subsets in the heart. They self-renew mainly through local proliferation. In *Cx3cr1* ^CreER-YFP^:*R26* ^Td^ mice, depleting resident macrophages during MI promoted adverse ventricular remodeling and exacerbated cardiac function (49). This result illustrates a protective role of resident macrophages. In contrast to resident subset, another study showed that the infiltration of monocyte-derived CCR2^+^ macrophages was required for adverse ventricular remodeling in pressure overload mice (50). In human, the analog phenomenon had also been reported (51).

**Figure 2:**
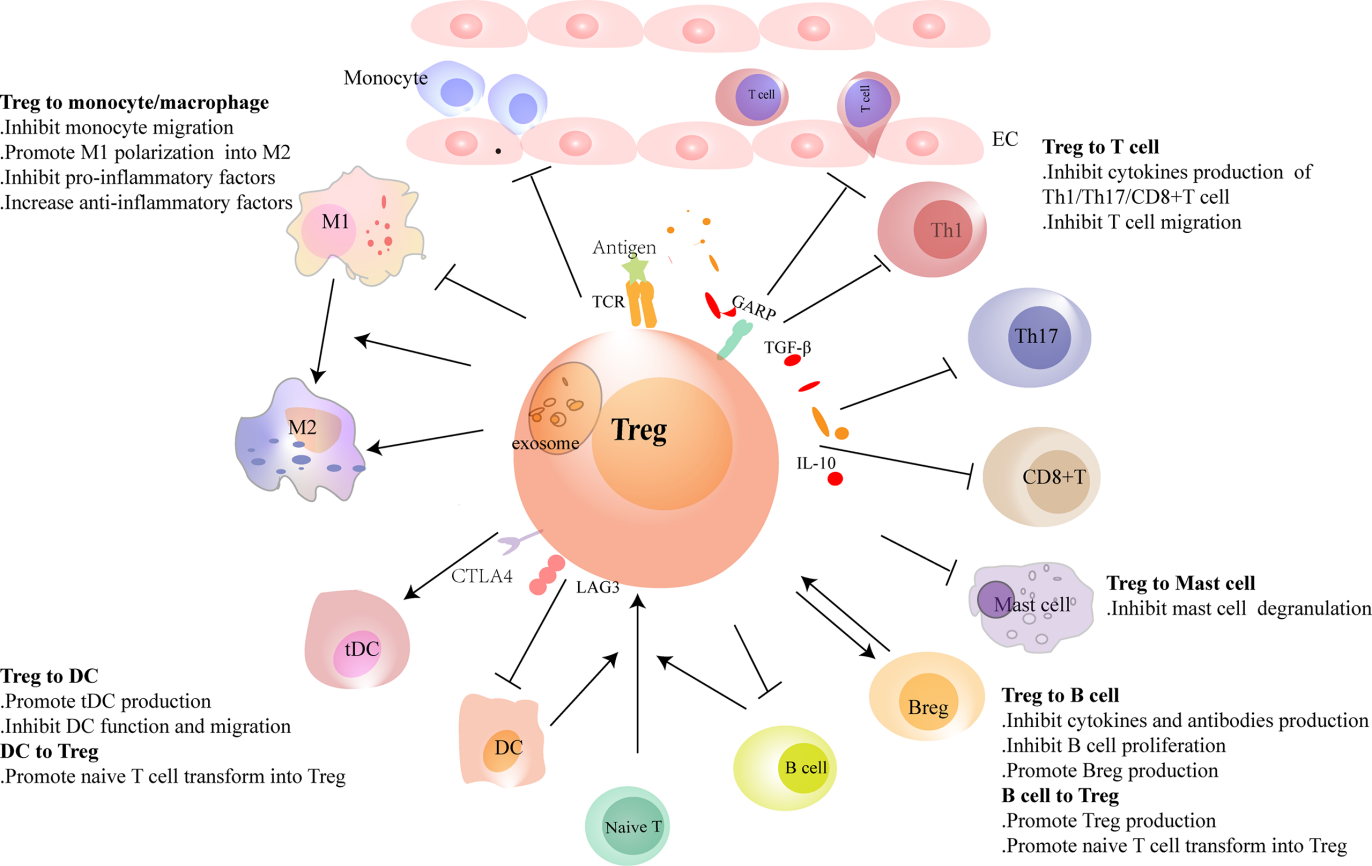
Regulatory T cells (Tregs) interact with immune cells. Tregs regulate the recruitment and functions of immune cells through cell-to-cell contact or cytokine or exosome secretion. Tregs reduce the migration of monocytes from the circulation and promote the polarization of monocytes/M1 macrophages to M2 macrophages. Conventional dendritic cells (cDCs) induce naive T cells to differentiate into Tregs in physiological homeostasis. However, in heart injury, Tregs assist in the generation of tolerance DC (tDCs), and tDCs induce Treg proliferation in turn. Tregs inhibit the migration of T cells and regulate the function of Th1, Th17, and CD8^+^ T cells and reduce the production of pro-inflammatory factors and antibodies in B cells. Tregs promote the proliferation of regulatory B cells (Bregs). B cells, including Bregs, promote Treg proliferation. In heart failure, the balance of Th1/Th2 and Th17/Treg shifts to Th1 and Th17, respectively. Tregs are dysfunctional in both number and function in heart failure. Their inhibitory functions in T cells and B cells are damaged.

Tregs play protective roles in ischemic heart disease, the main cause of HF. They regulate monocyte infiltration, reduce inflammation-related molecule secretion, and promote macrophage survival and M2 polarization. Weirather et al. (21) found that elimination of Tregs before MI increased the number of pro-inflammatory myeloid cells. The markers related to M1 macrophages were also elevated. These changes led to a poor prognosis in mice (21). In mouse I/R injury, the similar effects of Tregs had also been reported (52). IL-35, which is mainly expressed by Tregs, maintained the survival of CX3CR1^+^Ly6C^low^ macrophages and reduced cardiac rupture after MI in mice (53). Recently, it was reported that the exosomes derived from Tregs reduced the infarct size in mice by promoting the polarization of macrophages to M2 subset after MI (54). In *in vitro* experiments, the same effect of Tregs on the polarization of monocytes had also been described (55). Except for ischemic heart disease, hypertension and myocarditis are also important causes of HF. Adoptive transfer of Tregs ameliorated cardiac damage in Ang II-induced hypertension model and CVB3-related myocarditis model. They made a visible reduction of macrophages (28, 56) (**Figure 2** and **Table 1**). These results show that Tregs regulate the number and function of macrophages. However, it is still unclear whether Tregs influence the proliferation and/or polarization of macrophages locally in the lesion or just inhibit the recruitment of some specific monocytes selectively.

**Table 1:** The interaction between tregs and innate immune cells in HF.

Cell Type	Innate Immune Cells in HF	Interaction Between Tregs and Innate Immune Cells in HF
Monocyte/Macrophage	①Pro-inflammatory macrophages increased in the heart of HF mice. Splenectomy ameliorated cardiac remodeling and inflammation. Adoptive transfer of HF mouse splenocytes induced cardiac remodeling (46). ②Recruitment and local proliferation contributed to the expansion of macrophages in the remote myocardium of MI-induced HF. Inhibiting the recruitment of monocytes improved heart function in MI mice (47). ③Depletion of macrophages increased left ventricular dilatation and wall thinning in cryoinjury mice (48). ④Depletion of resident macrophages exacerbated cardiac function and promoted adverse cardiac remodeling in MI (49). ⑤Depletion of CCR2^+^ macrophages alleviated ventricular remodeling, dysfunction, and cardiac fibrosis on pressure overload mice (50). ⑥CCR2^+^ macrophages are a pro-inflammatory population and were associated with persistent LV systolic dysfunction in human HF (51).	①Tregs reduced the migration of monocytes and promoted the polarization of macrophages to M2 macrophages (21, 52, 54). ②Elimination of Tregs in I/R mice increased pro-inflammatory macrophages, accelerated ventricular dilation, and accentuated apical remodeling. Increasing Tregs in I/R mice reversed the effects (52). ③In Ang II-induced hypertension mice, adoptive transfer of Tregs reduced macrophages in the heart and ameliorated cardiac damage (28). ④Adoptive transfer of Tregs improved heart function in CVB3-related myocarditis (56).
Dendritic cell	①cDCs increased in the heart of HF mice. Splenectomy ameliorated cardiac remodeling and inflammation. Adoptive transfer of HF mouse splenocytes induced cardiac remodeling (46). ② Depletion of DCs deteriorated heart function and ventricular remodeling with increased pro-inflammatory cytokines and decreased anti-inflammatory cytokines in MI (57). ③G-CSF improved, but GM-CSF aggravated, early ventricular remodeling in MI rats (58).	①Tregs promoted the production of tDCs by secreting extracellular vesicles (59). ②The interaction of Tregs and DCs reduced costimulatory molecule expression on DCs and led to inadequate activation of effector T cells (60). ③cDC1s induced the production of self-tolerant Tregs. cDC2s presented heart antigens to effector T cells and promoted T-cell activation and polarization in MI (61). ④tDCs increased antigen-specific Tregs and produced beneficial effects in MI. *In vitro*, antigen-loaded DC or GM-GSF-stimulated DC induced the proliferation of Tregs (62–64).

Treg, regulatory T cell; HF, heart failure; MI, myocardial infarction; LV, left ventricular; DC, dendritic cell; cDC, classic DC; CCR, C-C motif Receptor; G-CSF, granulocyte colony-stimulating factor; GM-CSF, granulocyte-macrophage colony stimulating factor; I/R, ischemia-reperfusion; Ang, angiotensin; CVB3, coxsackievirus B3; tDC, tolerogenic DC.

Despite owning the fruitful results in animal research, rarely had a study on the direct interaction between Tregs and macrophages been done in HF patients. Whether macrophages in acute injury and chronic HF have different phenotypes and functions needs further study. Furthermore, we wondered if Tregs can still promote the M2 macrophage polarization in the end stage of heart injury. A study found that Tregs enhanced the efferocytosis of macrophages in inflammation-related diseases in mice. Whether this effect of Tregs is also existing in HF or not needs further investigation (65). Unlike the large number of macrophages, Tregs account for a small but indispensable part in cardiac injury. It reiterates that Tregs may synergize with macrophages through a cascade amplification effect. This effect may enhance the anti-inflammatory and pro-healing roles of Tregs.

### Regulatory T Cells Interact With Dendritic Cells in Heart Failure

DCs, which serve as the bridge between innate and adaptive immune cells, perceive the changes in the environment. They play important roles in immune tolerance and immune response. According to different sources, DCs are divided into myeloid DCs (mDCs) and plasmacytoid DCs (pDCs). mDC, which is also DC1, is derived from myeloid stem cells under the stimulation of granulocyte-macrophage colony-stimulating factor (GM-CSF). pDC, also known as DC2, is derived from lymphoid stem cells. Controlling helper T (Th) cell differentiation is the main function of DCs. DC1 produces IL-12 and induces the differentiation of Th cells into Th1 cells. However, DC2 hardly produces IL-12 and promotes the differentiation of Th cells into Th2 cells.

The number of DCs increased in the hearts in both acute injury and HF. It indicated that they may have distinct functions in different phases of heart injury (46, 66, 67). DCs ameliorated left ventricular remodeling after MI. Elimination of DCs by using CD11c^DTR^ mice worsened ventricular remodeling and function (57). However, treating MI rats with granulocyte colony-stimulating factor (G-CSF) or GM-CSF revealed that G-CSF improved but GM-CSF aggravated ventricular remodeling (58). These controversial results may be associated with the expansion of different DC subsets in the heart. Cross-priming DCs have the ability to activate both CD4^+^ T cells and CD8^+^ cytotoxic T cells. Recently, using *Clec9a*-depleted mice that were deficient in DC cross-priming, Forte E. et al. (68) found that in ischemic HF, cross-priming DCs accumulated in hearts and contributed to the exacerbation of post-ischemic inflammatory damage. These results may remind us of the different roles of DCs in the process of heart injury.

Although majority of studies found that the total number of DCs in peripheral blood is elevated in HF patients, the results are inconsistent (69–73). The differences in severity and etiology of HF may be responsible for the discordant results. It reminds us that more detailed and reasonable classification of HF is needed for further clinical research.

The interaction between Tregs and DCs has not been fully elucidated. Tolerogenic DC (tDC), which is blunt to certain antigens, acts as a protective subset in many immune-related diseases. Tregs promoted the production of tDCs by secreting extracellular vesicles containing miR-150-5p and miR-142-3p *in vitro* (59). Except for generating tDCs, Tregs also influence the maturation and function of DCs. A few studies showed that the interaction between Tregs and DCs reduced the costimulatory molecule expression on DCs. These defective DCs led to inadequate activation of effector T cells *in vivo* (60, 74). Similarly, after coculturing with CD4^+^CD25^+^ Tregs but not CD4^+^CD25^-^ T cells, DCs reduced their maturation and their ability in antigen-presenting was also damaged (75).

DCs have direct effects on Tregs. In homeostatic conditions, immature DCs induced the anergy of self-reactive T cell and promoted the differentiation of naive T cells into autoantigen-specific Tregs. This process was related to cDC1 (61, 76). In addition to cDC1, tDCs can also promote the formation of antigen-specific Tregs during injury. This may be a treatment strategy for immune-related diseases. Stimulating bone marrow-derived DCs with TNF-α and cardiac lysates from MI mice induced the generation of tDCs. Adoptive transfer of these tDCs into MI mice increased antigen-specific Tregs in lymph nodes, spleens, and hearts. Finally, they achieved a beneficial effect on ventricular remodeling (62). Similarly, the effects of tDCs in increasing the number of Tregs had also been reported in autoimmune myocarditis and chronic Chagas disease mice (63, 64). DCs enhanced Treg functions in inhibiting the proliferation and accumulation of effector T cells likewise, which avoid excessive autoimmune response caused by cardiac injury (77). Just like *in vivo*, antigen-loaded DCs, especially mature DCs, stimulate the proliferation of Tregs directly *in vitro* (78) (**Figure 2** and **Table 1**).

Although the changes in the number of DCs had been described in a series of clinical studies of HF, there is a lack of description on the direct interaction between Tregs and DCs *in vivo*. Some new breakthroughs are urgently needed.

### Regulatory T Cells Interact With Effector T Cells in Heart Failure

T cells are heterogeneous groups that output from the thymus. T cells consist of CD4^+^ T cells and CD8^+^ T cells. CD4^+^ T cells are the main T-cell subset in heart injury, and the classification of CD4^+^ T cells is mainly based on the cytokines they produced. Common types of CD4^+^ T-cell subsets include Th1 cells [producing interferon (IFN)-γ], Th2 cells (producing IL-4, IL-5, and IL-13), Th17 cells (producing IL-17), and Tregs (producing IL-10, TGF-β, and IL-35). Based on the constitution of TCR, T cells can also be divided into αβT cells (including CD8^+^ T cells, Th1, Th2, and Th17 cells) and γδT cells.

T cells infiltrated into the damaged heart and their deficiencies in the number and function are involved in the pathophysiological process of HF. CD4 or MHC-II knockout mice are lack of CD4^+^ T cells. Experiments using these mice showed that CD4^+^ T cells were indispensable for proper collagen deposition in the infarct area (79). Myosin heavy chain alpha (MYHCA) is the main autoantigen released during MI. By transferring MYHCA_614-629_-specific CD4^+^ T cells to recipient mice, Rieckmann et al. (80) found that these cells accumulated in mediastinal lymph nodes and hearts after MI. Surprisingly, a large number of the transferred cells transformed into Tregs and obtained a distinct pro-healing effect (80). However, eliminating CD4^+^ T cells from the fourth week after ligation reduced the infiltration of CD4^+^ T cells in hearts and rescued the left ventricular dilatation in HF mice. Adoptive transfer of heart-infiltrating CD3^+^ T cells or splenic CD4^+^ T cells from HF mice to naive recipient mice led to a damaged left ventricular function (81). These results indicate a plastic function of T cells in acute injury and HF. It may be related to the different activation and transcription characteristics of T cells at different stages. As the main source of IFN-γ, Th1 cells are harmful to the ischemic heart in mice (82). IL-17A produced by γδT cells exacerbated left ventricular remodeling in both MI and myocardial I/R injury mice (83–85). Although cytotoxic CD8^+^ T cells played a negative role in MI mice, CD8^+^AT2R^+^ T cells secreted IL-10 and potentially facilitated wound healing after MI (21, 86).

T-cell infiltration had also been described in endocardial biopsies of patients with inflammatory DCM. It mainly consisted of Th1, Th2, and Treg cells. Moreover, the correlation between the characteristics of T-cell receptor V-beta (TRBV) and etiology had also been described (87). Recently, Tang et al. (88) found an aggregation of T cells in the hearts of ischemic HF patients, which included a large number of CD4^+^ T cells and CD8^+^ T cells. Furthermore, they described an oligoclonal characteristic of heart tissue-specific TCRs that is different from peripheral blood T cells (88). These results suggest an antigen-related activation of T cells.

Except for the changes of a certain T-cell population, the imbalance between T-cell subsets had also been observed in HF. Eight weeks after MI, a reduced Th1/Th2 ratio and an increased Th17/Treg ratio were observed in mice (81). Exercise, catechin, fenofibrate, and inhibition of micro-RNA155 had been reported to reverse the imbalance of Treg/Th17 ratio in mice (89–92). In human, HF was usually accompanied by immune activation. The balance of Th1/Th2 and Th17/Treg in circulation shifted to Th1 and Th17, respectively (40, 93–96). What factors drive these shifts and whether these changes are caused by, or lead to, adverse ventricular remodeling and HF need to be established.

The research on Tregs in suppressing the infiltration and function of T cells is abundant in the heart. In MI, Treg supplementation reduced the number of CD3^+^ T cells in the heart. On the contrary, ablation of Tregs increased the absolute counts of CD4^+^ T cells and CD8^+^ T cells in mice (21, 22). In *in vitro* experiments, Tregs obtained from the spleens of MI mice were defective in inhibiting the function of conventional T cells. However, recently, we found that Tregs infiltrating into MI hearts had increased expression of CTLA-4 and KLRG-1 in mice. It indicates an enhanced inhibitory capacity of heart Tregs. These results may indicate a different function of tissue Tregs and lymphoid Tregs (20, 97). In a previous study, we also showed that Tregs inhibited the response of cytotoxic CD8^+^ T cells in mice after MI (22). Adoptive transfer of Tregs attenuated cardiac remodeling by reducing IFN-γ expression in MI mice (98). Exosomes secreted by Tregs containing micro-RNAs, such as Let-7d, regulate the function of a variety of immune cells in mice. They inhibited the proliferation and cytokine production of Th1 cells (99). Whether this mechanism is also involved in HF needs further exploration. As a subset of Tregs, CD4^+^CD25^+^GARP^+^ Tregs obtained from the peripheral blood of DCM patients were dysfunctional in suppressing the proliferation of Tresp cells (CD4^+^CD25^-^GARP^-^). It may explain the hyperinflammatory state of DCM patients (39) (**Figure 2** and **Table 2**). Study on the direct effect of Tregs on Th17, Th2, or newly discovered Th9 and Th22 cells in HF is scarce, which needs more research.

**Table 2:** The interaction between tregs and adaptive immune cells in HF.

Cell Type	Adaptive Immune Cells in HF	Interaction Between Tregs and Adaptive Immune Cells in HF
T cell	① CD4^+^ T cell-deficient MI mice displayed aggravated ventricular dilation and greater mortality and heart rupture rates (79). ②Elimination of CD4^+^ T cells reduced inflammatory cell infiltration and reduced left ventricular dilatation in HF mice. Transferring heart-infiltrated CD3^+^ T cells or splenic CD4^+^ T cells from HF mice to naive recipient mice resulted in left ventricular dysfunction (81). ③ Adoptive transfer of heart antigen-specific MYHCA_614-629_ CD4^+^ T cells acquired a Treg phenotype and showed pro-healing effects (80). ④ Th1 cells is the main source of IFN-γ. IFN-γ aggravated wound healing by influencing the functions of fibroblast (82). ⑤ γδT cells is the major source of IL-17A. IL-17A exacerbated left ventricular remodeling after heart injury in both MI and I/R (83–85). ⑥ CD8^+^AT2R^+^ T cells contributed to maintaining cardiomyocyte viability and reduced ischemic heart injury by increasing IL-10 and reducing IL-2 and IFN-γ expression (86). ⑦ Tregs became Th1-like cells with antiangiogenic and profibrotic effects and participated in adverse ventricular remodeling in HF (31).	①Adoptive transfer of Tregs attenuated cardiac remodeling by reducing IFN-γ expression in MI (98). ②Exosomes produced by Tregs inhibited the proliferation and cytokine secretion of Th1 cells that may be involved in the regulation of heart damage (99). ③Tregs inhibited the response of CD8^+^ T cells after MI by reducing pro-inflammatory factors of CD8^+^ T cells (22). ④Tregs from HF patients were dysfunctional in suppressing the function of responder T cell (Tresp) (39).
B cell	①B cells induced Ly6C^+^ monocyte infiltration by producing CCL7. Elimination of B cells improved heart function after MI (100). ②B cells assisted in the increase of T cells and DCs in pericardial adipose tissue. CB2^-/-^ mice showed deteriorated ventricular remodeling after MI, and pericardial adipose tissue removal reversed the effect (101). ③Rituximab or antibody-dependent B-cell deletion reversed myocardial hypertrophy and improved cardiac function (102, 103). ④ DCM patients presented a high frequency of CD19^+^ B cells, and the percentage of TNF-α producing B cells increased obviously (104). ⑦Bregs were deficient in number and function in DCM patients (105). ⑧Bregs were enriched in pericardial adipose tissue and attenuated post-MI inflammation and improved the outcome of MI (106).	①CD4^+^LAP^+^ Tregs in the peripheral blood of DCM patients were dysfunctional. Their abilities in inhibiting B-cell proliferation and antibody production were impaired (107). ② Bregs induced the expansion of Tregs, and Tregs also increased the number of Bregs in turn (108). ③ B cells induced the production of Tregs (109, 110).

Treg, regulatory T cell; MI, myocardial infarction; Th, helper T cell; IFN, interferon; IL, interleukin; AT2R, angiotensin Ⅱ type 2 receptor; CCL, chemokine C-C motif ligand; DC, dendritic cell; CB, cannabinoid receptor; DCM, dilated cardiomyopathy; TNF, tumor necrosis factor; Breg, regulatory B cell; Tresp, responder T cell; LAP, leucine aminopeptidase; CD, cluster of differentiation.

### Regulatory T Cells Interact With B Cells in Heart Failure

As another adaptive immune cell subset, B cells include B1 cells and B2 cells. B1 cells (including B1a and B1b) produce natural antibodies without any stimulation of exogenous antigens during prenatal life. However, B2 cells, including follicular B cells and marginal zone B cells, are generated postnatally and are unusually stimulated by exogenous antigens. Except for the different sources, B1 cells and B2 cells have specific markers separately. B1 cell is CD19^+^CD11b^+^IgM^+^ subset in mice, and B1a also has a high expression of CD5, but B1b is CD5 negative. However, in human, B1 cells are CD20^+^CD27^+^CD38^low/int^CD43^+^. B2 cells are CD19^+^CD11b^-^ (111, 112).

B cells accumulate in the heart during the acute phase of heart injury. A study showed that B cells induced the infiltration of Ly6C^+^ monocytes by producing CCL7. Elimination of B cells improved heart function after MI in mice (100). Moreover, B cells assisted the increase of a variety of immune cells, including T cells and DCs, in pericardial adipose tissue. Research using cannabinoid receptor CB2 knockout (CB2^-/-^) mice, which have an increased number of B cells, showed deteriorated ventricular remodeling after MI (101). Deleting B cells with anti-CD22 reduced the pro-inflammatory cytokine production. It alleviated heart fibrosis in heart hypertrophic mice induced by Ang II (102). In HF, blocking CD20 on B cells with rituximab reversed myocardial hypertrophy and improved cardiac function in transverse aortic constriction mouse models (103). Compared with healthy controls, Yu et al. (104) found a high frequency of CD19^+^ B cells in the peripheral blood of DCM patients. Furthermore, they reported that the percentage of TNF-α, but not IL-10, producing B cells increased perceptibly (104). All these results suggest a negative role of B cells in HF-related diseases. Mechanistically, besides producing antibodies, B cells also secrete abundant cytokines, such as IL-1, IL-6, TNF, TGF-β, and IL-10. They can also interact with other immune cells directly. Apart from Tregs, a group of IL-10^+^ B cells with regulatory function called regulatory B cells (Bregs) had also been discovered (113). In pericardial adipose tissue, CD5^+^ Bregs were enriched and attenuated the inflammation response after MI in mice (106). According to our research, Bregs in DCM patients were deficient in number. Their effect in inhibiting the function of conventional T cells was also damaged (105).

Tregs regulate the functions of B cells in multiple ways. They interacted with B cells directly and inhibited antibody production (114). Moreover, Tregs participated in the peripheral tolerance by restraining the proliferation of autoreactive B cells and promoting their apoptosis (115). However, our previous study showed that CD4^+^LAP^+^ Tregs in the peripheral blood of DCM patients were dysfunctional. Their abilities in inhibiting B-cell proliferation and antibody production were impaired compared to healthy controls. This change in B cells may lead to immune disorders in HF (107).

Besides being the target of Tregs, B cells can also act aggressively in Treg proliferation. Experiments showed that B cells, including Bregs, promoted Treg proliferation, which may associate with homeostasis maintenance (108–110) (**Figure 2** and **Table 2**). Studies on the interaction between Tregs and B cells are fruitful. However, it is poorly understood during HF, and a more in-depth investigation is needed.

## Regulatory T Cells Interact With Heart Failure-Related Parenchymal Cells

### Regulatory T Cells Interact With Cardiomyocytes in Heart Failure

CMs are striated self-beating and cylindrical rod-shaped muscle cells that fundamentally govern the function of the myocardium. Most CMs are rich in myofibrils. They are tightly connected and form a muscle fiber network to participate in the systolic and diastolic functions of the heart. However, there is a small part of CMs that lacks myofibrils. They are highly self-disciplined and take part in cardiac electrical conduction. CMs are vulnerable to hypoxia. Once damaged, necrotic CMs release damage-associated molecular patterns (DAMPs) and cytokines that are associated with acute inflammation response and ventricular remodeling. Transfusion of Tregs or expanding endogenous Tregs by IL-2C reduced the apoptosis of CMs and ameliorated cardiac function in MI mice (22, 26). In addition to the antiapoptotic effect, Tregs also promote the proliferation of CMs directly according to recent research. Zacchigna et al. (116) found that Tregs promoted the survival of CMs in MI mice during pregnancy by generating CST7, TNFSFL1, IL33, FGL2, MATN2, and IGF2. Another study also revealed that within 1 week after birth, Tregs aggregated in hearts after injury and played a protective role in ameliorating cardiac fibrosis in mice. Single-cell RNA sequencing showed that Tregs promoted the proliferation of CMs in a paracrine manner. They produced regeneration-related molecules, including CCL24, Areg, and GAS6 (117). These results give rise to the tissue regeneration function of Tregs in the heart, which had been confirmed in injured muscle and skin (16, 17). *In vitro*, Tang et al. (22) found that Tregs mitigated the apoptosis of neonatal rat CMs induced by lipopolysaccharide (LPS). This effect was cell-to-cell contact dependent. IL-10 but not TGF-β also participated in the process (22). In addition to maintaining the number of viable CMs, Tregs may directly restrict the pro-inflammatory cytokine secretion, such as IL-1β and TNF-α in CMs. A deeper understanding of the protective mechanism of Tregs in CMs may provide a new strategy for the treatment of HF.

### Regulatory T Cells Interact With Fibroblasts in Heart Failure

Timely activation and proliferation of cardiac fibroblasts are important for effective repair after heart injury. Myofibroblasts that transformed from fibroblasts produce growth factors, cytokines, chemokines, and ECM components. They are involved in wound healing and facilitate the recruitment and activation of immune cells. Single-cell RNA sequencing of cardiac interstitial cells revealed that fibroblasts are heterogeneous populations in mice after MI. They had either profibrotic or antifibrotic signature and participated in the heart’s responses to injury (118). Fibroblasts are critical to maintain heart integrity in injury. However, excessive activation of fibroblasts or diminished apoptosis of myofibroblasts unusually means poor repair after heart injury (119). A recent study reported a novel function of fibroblasts beyond tissue repair and fibrosis. Fibroblast-specific protein 1 (FSP1) expressing fibroblasts participated in angiogenesis in mice and played a protective role after MI (120).

Tregs can interact with fibroblasts directly. Coculture experiment of fibroblasts and splenic Tregs showed that Tregs modulated the phenotype of fibroblasts and reduced the expression of alpha-smooth muscle actin (α-SMA) and matrix metalloproteinase-3. Therefore, attenuated the contraction of fibroblast-populated collagen pads (52). Yet, our latest research found that a unique population of Tregs producing SPARC accumulated in the heart. They reduced heart rupture by increasing the production of collagen III in fibroblasts after MI in mice. *In vitro*, coculture of fibroblasts with SPARC-overexpressing Tregs confirmed the result (20). These results indicate that Tregs inhibit the excessive activation of fibroblasts. This may give an explanation of the protective role of Tregs in the non-infarct area during ischemic heart injury. However, in the infarct area, promoting collagen production and deposition are the primary functions of Tregs.

### Regulatory T Cells Interact With Endothelial Cells in Heart Failure

Endothelial cells (ECs) are essential in heart homeostasis and tissue repair. They influence both vascular and immune systems. Cardiac ECs are involved in the formation of vessels and regulate the integrity of the vascular. Injury-activated ECs participate in inflammation response by producing cytokines, chemokines, and growth factors, such as IL-6, P-selectin, E-selectin, vascular cell adhesion molecule (VCAM)-1, and intercellular adhesion molecule (ICAM)-1. Hypoxia induced the generation of vascular growth factors and promoted the activation of ECs, which induced vessel formation in ischemic and hypertrophic hearts (121). Therefore, ECs are considered immunoregulatory cells by some immunologists, although they have no direct functions on phagocytosis, antibody production, and cellular immunity.

**Figure 3:**
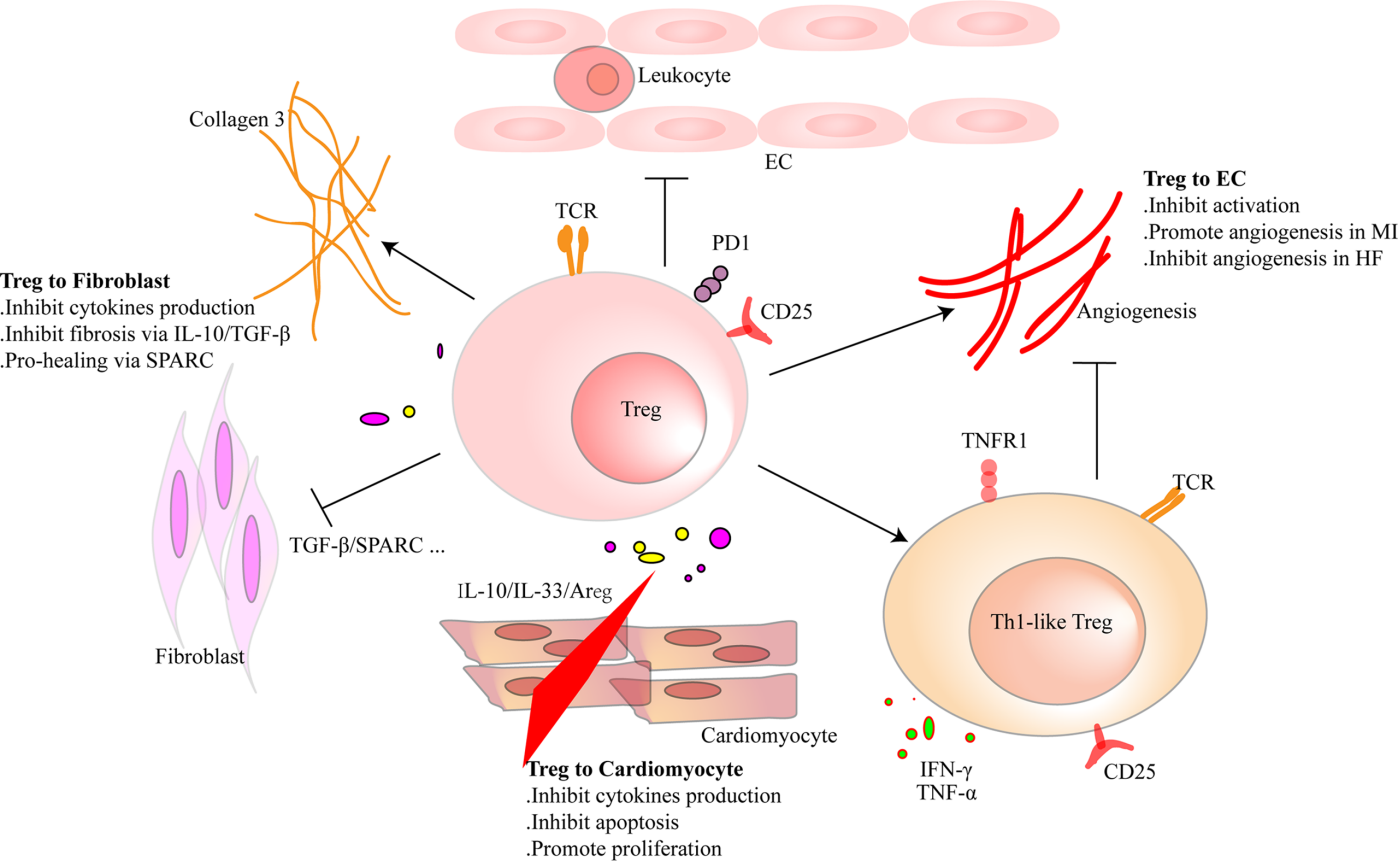
Regulatory T cells (Tregs) interact with cardiac parenchymal cells. Tregs regulate the functions of cardiac parenchymal cells through cell-to-cell contact or molecule secretion. For cardiomyocytes, Tregs inhibit pro-inflammatory factor production and reduce the apoptosis of cardiomyocytes after heart injury in adult mice. Moreover, they directly promote the proliferation of cardiomyocytes in injured mice of a week old or pregnant. Tregs inhibit pro-inflammatory factor production of fibroblasts and promote collagen III synthesis in fibroblasts through secreted protein acidic and rich in cysteine (SPARC) secretion in the infarcted area. Tregs inhibit fibrosis of the interstitial zone by regulating fibroblast activation. Tregs inhibit the activation of endothelial cells (ECs) and reduce the migration of leukocytes. In the early stage of heart injury, Tregs promote angiogenesis. However, dysfunctional pro-inflammatory Tregs in heart failure inhibit the effect.

Regulating the activation of ECs and angiogenesis and modulating leukocyte migration are the main effects of Tregs on ECs. Pulmonary arterial hypertension is one of the causes of right ventricular HF. In mice, elimination of Tregs decreased the production of several vascular protection-related proteins in ECs, such as cyclooxygenase 2 (COX-2), prostaglandin I2 synthase (PTGIS; prostacyclin synthase), programmed death ligand 1 (PD-L1) and heme oxygenase 1 (HO-1) and worsened pulmonary hypertension. However, the protective effect appeared again by transferring Tregs *in vivo*. *In vitro*, coculture of human heart microvascular ECs with Tregs also increased these protective proteins (122). Furthermore, Tregs inhibited the activation of human umbilical vein endothelial cells (HUVECs) that was induced by oxidized low-density lipoprotein and LPS *in vitro*. They reduced the expression of VCAM-1, monocyte chemoattractant protein monocyte chemotactic protein-1, and IL-6 in ECs (123). Similarly, fine particles and vasoactive substances, such as Ang II, also act as EC-activating factors. However, Tregs inhibited the activation of ECs in these situations (124, 125). Effective angiogenesis prevents HF by reducing adverse ventricular remodeling. ECs are the main members in the process (126). Transferring Tregs improved the prognosis of MI in mice by increasing the production of small capillaries (<10 mm) (97). In HF mice, Tregs were significantly increased. However, they transformed into Th1-like pro-inflammatory cells with the capacity to produce IFN-γ, TNF-α, and tumor necrosis factor receptor (TNFR)1. Compared with naive Tregs, they exhibited an antiangiogenic effect when cocultured with rat coronary ECs (31). Different functions of Tregs between early stage of MI and HF explain the temporal and spatial heterogeneity of Tregs. During inflammation, CD73 on the Tregs protected the integrity of the vascular endothelium and reduced leukocyte transcellular migration (127). An *in vitro* study reported that iTregs, but not nTregs, passed through the single layer of endothelium and inhibited the activation of ECs. This made a reduced migration of effector T cells and attenuated inflammatory response (128). After coculturing with HUVECs, Tregs obtained a stronger suppression function on effector T cells. This effect was related to the interaction of PD-1 on Tregs and PD-L1/PD-L2 on activated ECs (129). In human, HLA-DR^+^ ECs promoted Tregs’ proliferation through the surface marker CD54 on ECs (130). The interaction between Tregs and ECs is abundant. Targeting ECs may be a chance for HF patients, and more research is needed for better clinical treatment. The crosstalk between Tregs and parenchymal cells is complex and needs more investigation (**Figure 3** and **Table 3**).

**Table 3:** The interaction between tregs and parenchymal cells in HF .

Cell Type	Parenchymal Cells in HF	Interaction Between Tregs and Parenchymal cells
Cardiomyocyte	Cardiomyocyte is the main cell type in the heart. It participates in heart contraction and produces pro-inflammatory factors under stress conditions.	①Tregs reduced pro-inflammatory factor production of hypoxic cardiomyocytes *in vitro* (22). ②Tregs reduced the apoptosis of hypoxic cardiomyocytes and ameliorated cardiac function in MI mice (22, 26). ③Tregs promoted cardiomyocyte proliferation directly (116, 117).
Fibroblast	Fibroblast is the main cell type in the heart. During heart damage, fibroblasts are activated and participate in tissue repair and adverse ventricular remodeling. ①Fibroblasts in the heart were heterogeneous populations and constituted multiple subsets. FSP1-expressing fibroblasts participated in promoting angiogenesis and played a protective role in MI (118, 120). ②Excessive activation of fibroblasts and diminished apoptosis of myofibroblasts participated in poor repair after heart injury (119).	①Tregs accumulated in MI heart produced SPARC, which reduced heart rupture by increasing the production of collagen III in fibroblasts. *In vitro*, coculture of fibroblasts with SPARC-overexpressing Tregs had the same effect (20). ②*In vitro*, Tregs reduced the expression of α-SMA and MMP3 in fibroblasts and attenuated the contraction of fibroblast-populated collagen pads (52).
Endothelial cell (EC)	Cardiac ECs participate in the regulation of vascular integrity and have dual effects in coagulation and anti-coagulation. ①Hypoxia promoted activated ECs to form vessels in ischemic and hypertrophic hearts (121).	①Elimination of Tregs decreased the production of vascular protective related proteins in ECs and worsened pulmonary hypertension. In coculture of human heart microvascular ECs with Tregs, these proteins increased (122). ②Tregs inhibited the activation of ECs induced by ox-LDL, LPS, fine particles, and vasoactive substances (123–125). ③Adoptive transfer of Tregs induced an increase in small capillaries in MI (97). ④Tregs became Th1-like cells with an antiangiogenic effect in HF. This effect was related to the expression of TNFR1 on Tregs (31). ⑤Tregs protected the integrity of vascular endothelium and reduced the leukocyte transcellular migration (127). ⑥In coculture of Tregs with HUVECs, the suppressive function of Tregs on effector T cells increased (129). ⑦HLA-DR^+^ ECs promoted Treg proliferation through the surface marker CD54 (130).

FSP, fibroblast specific protein; MI, myocardial infarction; EC, endothelial cells; SPARC, secreted protein acidic and rich in cysteine; SMA, smooth muscle actin; MMP, matrix metalloproteinase; ox-LDL, Oxidation Low Lipoprotein; LPS, lipopolysaccharide; HF, heart failure; TNFR, tumor necrosis factor receptor; HUVEC, human umbilical vein endothelial cell; HLA, human leukocyte antigen.

Apart from the cells mentioned above, the changes in neutrophils, natural killer (NK) cells, eosinophils, and mast cells had also been observed in heart injury. Tregs reduced the infiltration of neutrophils after acute heart injury and ameliorated heart function. Just like what was observed in bone marrow transplant tolerance in mice, Tregs may also promote the education of NK cells (22, 131). Moreover, suppressing mast cell degranulation is another function of Tregs. However, whether this mechanism is involved in HF needs further research (132). Although Liu et al. (133) had reported a protective role of eosinophils in MI recently, whether Tregs help the effect of eosinophils deserves further study.

## Conclusions and Perspectives

The crosstalk between Tregs and immune cells or parenchymal cells is complex. Numerous preclinical studies on Tregs have been conducted in type 1 diabetes, organ transplantation, and autoimmune diseases. Evidence supported that Tregs were safe in protecting patients of autoimmune diseases and organ transplant rejection. In recent years, researchers found that antigen-specific Tregs and engineered Tregs were more efficient in the treatment of disease. They had stronger suppression function or more precisely accumulated after injury compared with polyclonal Tregs (134). However, it is worth noting that treatment with Tregs may cause systemic immune disorders sometimes. Manufacturing Tregs for clinical applications and monitoring the biological functions of transferred Tregs *in vivo* are needed to be addressed firstly. Furthermore, finding ways for commercialization of Treg-based therapy is also a great challenge.

Despite the difficulties, next-generation sequencing, including bulk sequence and single-cell sequence, gives us the chance to explore the functions of immune and parenchymal cells in heart development and injury. More importantly, single-cell sequencing helps us to map the development trajectories of cells by in-depth analysis of the transcriptome characteristics of single cells. The characteristics of individual Tregs had been described in atherosclerosis, myocarditis, MI, and pressure overload-induced HF in mice (4, 5, 20, 135). We also reported the differences in transcriptome characteristics between heart Tregs and splenic Tregs after MI and described an oligoclonal feature of heart Tregs (20). All the results assisted us to identify the subset of Tregs that is beneficial in tissue repair or prognosis of HF.

Different pathophysiological changes exist in acute heart injury and HF. What needs to be solved urgently now is to dynamically describe the transcriptomic and TCR characteristics of Tregs along the development of HF. Based on this, modifying Tregs, such as generating heart injury-related CAR-Treg or constructing Treg with heart antigen-specific TCR, may inhibit excessive immune responses and regulate the functions of immune or parenchymal cells more powerfully. Compared with total Tregs, application of specific molecules of Tregs *in vivo*, such as some cytokines or transcription factors of heart Tregs, may be another potential choice. We believe that individualized treatment of HF will come true eventually with the development of biotechnologies. Describing the characteristics of the target cells of Tregs in HF in detail and understanding the mechanisms of their interactions will help us choose the most suitable method in the individualized therapy of HF.

In this review, we summarized the latest studies on Tregs in the early stage of heart injury and HF. At the same time, we described the interactions between Tregs and their target cells in HF and HF-related diseases, which will help us form a big picture of the inflammatory responses related to HF. Combining the latest technology methods with the basic knowledge of Tregs would contribute to the individualized treatment of HF. Taking different interventions based on different stages of heart injury may maximize the benefits of patients.

## Author Contributions

YL and NX conceptualized this review, decided on the content, and wrote the manuscript. YL prepared the tables and figures. XC revised this review. All authors contributed to the article and approved the submitted version.

## Funding

This work was supported by grants from the National Natural Science Foundation of China [No. 81770503 to NX; 81900451 to YL].

## Conflict of Interest

The authors declare that the research was conducted in the absence of any commercial or financial relationships that could be construed as a potential conflict of interest.

## Publisher’s Note

All claims expressed in this article are solely those of the authors and do not necessarily represent those of their affiliated organizations, or those of the publisher, the editors and the reviewers. Any product that may be evaluated in this article, or claim that may be made by its manufacturer, is not guaranteed or endorsed by the publisher.
